# Photodynamic therapy dosimetry: current status and the emerging challenge of immune stimulation

**DOI:** 10.1117/1.JBO.30.S3.S34118

**Published:** 2025-12-19

**Authors:** Brian C. Wilson, Lothar Lilge, Robert A. Weersink, Layla Pires

**Affiliations:** a University Health Network, Princess Margaret Cancer Research Institute Toronto, Ontario, Canada; b University of Toronto, Department of Medical Biophysics, Toronto, Ontario, Canada; c University of Johannesburg, Laser Research Centre, Johannesburg, South Africa; d University of Toronto, Department of Radiation Oncology, Toronto, Ontario, Canada; e University of Toronto, Institute of Biomedical Engineering, Toronto, Ontario, Canada; f Texas A&M University, Department of Biomedical Engineering, College Station, Texas, United States

**Keywords:** photodynamic therapy, dosimetry, photochemical immune stimulation, oncology

## Abstract

**Significance:**

Addressing the challenges of accurate dosimetry in photodynamic therapy has motivated some of the earliest work in tissue optics, which then enabled the broader development of biomedical optics. Inadequate use of dosimetry-informed treatments may contribute to heterogeneity in tumor response and variable clinical outcomes that need to be addressed.

**Aim:**

This perspective paper seeks to understand the current status of photodynamic therapy (PDT) dosimetry in preclinical and clinical applications and identify opportunities for improvement. We also identify the “elephant in the room” of photodynamic immune stimulation that presents additional dosimetry challenges and opportunities.

**Approach:**

The origins of PDT dosimetry based on biophysical metrics are considered, and major scientific and technological advances that have underpinned biological and clinical studies are highlighted. The question is posed: “Has the inadequacy of dosimetry for PDT been a major factor in this treatment not achieving widespread adoption into clinical practice, particularly in oncology?” It may be the case that, in the clinic and also frequently in preclinical (especially, *in vivo*) research, PDT dosimetry is often necessary, occasionally used, sometimes effective, and rarely sufficient. The rapid emergence of research on PDT immune stimulation poses existential challenges for PDT dosimetry as practiced to date, which is based on purely biophysical considerations, and possible approaches are suggested that incorporate immunological factors.

**Results:**

Different clinical situations require different PDT dosimetry approaches, depending on medical complexity and technical dosimetry requirements.

**Conclusions:**

This article is not a comprehensive review, but rather intended to recognize past advances and current limitations, and to stimulate discussion of future directions in PDT dosimetry. Inadequate dosimetry may be a potential impediment to PDT adoption and may have contributed to the failure of some previous and ongoing clinical trials.

## 1 Introduction

“…*less exact methods relied on specifying the drug dose and delay after injection and on the irradiance at the surface. …. The new formulation should permit closer control of the effective absorbed dose and better clinical results*…”, Dan Doiron and Ed Profio, 1987.^1^

Photodynamic therapy (PDT), using compounds (photosensitizers) activated by light to generate short-lived cytotoxic species (such as singlet oxygen, O12), has been used to treat a variety of localized solid tumors across different organ sites.^2^^,^^3^ The development of the canonical “first-generation” photosensitizer Photofrin™, along with emerging clinical lasers and fiberoptic light delivery, launched the modern era of PDT in the late 1970s.^4^ The field has since expanded in several directions:^5^ new synthetic photosensitizers with improved photodynamic and, in some cases, also fluorescence properties for tumor localization: active targeting of photosensitizers to improve tumor specificity; an expanded range of light sources and delivery technologies, together with dosimetry techniques and devices (the topic at hand); elucidation of the complex photobiological mechanisms of action and cell-death pathways involved in PDT; the use of nanoparticles to replace molecular photosensitizers and/or as photosensitizer delivery vehicles; radiodynamic therapy (RDT) using ionizing radiation as the activating energy source to overcome the limited tissue penetration of light; and, most recently, recognition and exploitation of anti-tumor immune stimulation by PDT to increase tumor response and impact metastasis. The last advance can extend PDT beyond being a purely localized treatment.

PDT has several biological and resulting clinical advantages compared with other cancer treatments:

• low systemic toxicity, apart from prolonged skin photosensitization with first-generation photosensitizers
• lack of induced resistance to treatment so that treatments can be repeated if only a initial response is achieved
• compatibility with other treatments such as radiotherapy and surgery, allowing adjuvant of combination protocols
• good preservation of normal host tissue structure and function
• multiplicity of cell-death pathways that can be exploited for maximum clinical efficacy
• evidence for significant PDT-induced stimulation of the innate and adaptive immune systems that can contribute to the eradication of the primary treated tumor, reduce tumor progression, and impact metastasis.

**Fig. 1:**
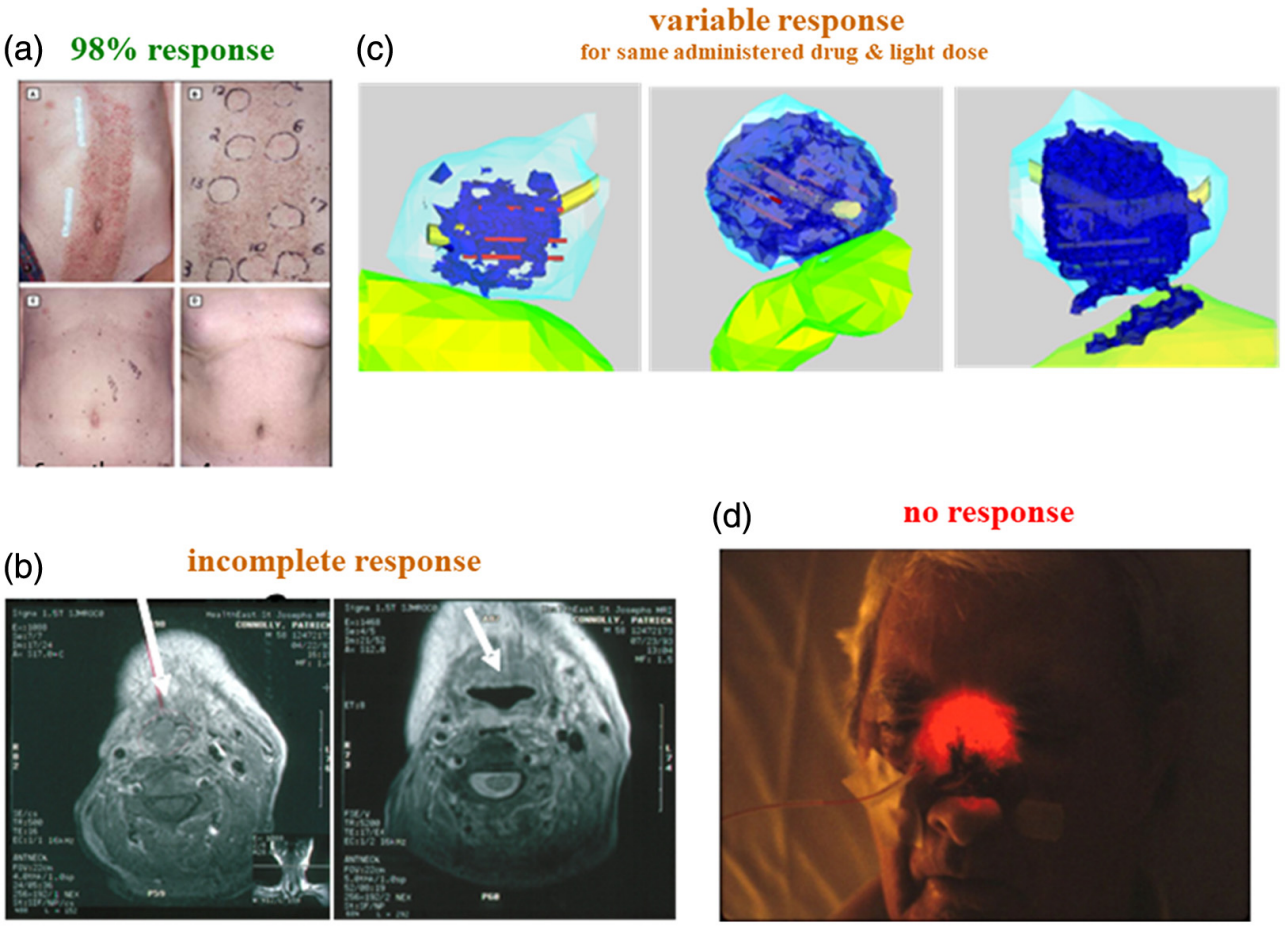
Examples of clinical cases showing large variability in response of solid tumors treated by PDT. (a) Photos of extensive basal cell carcinomas induced by prior radiation therapy: before (top left), during course of treatment showing the individual patches targeted (top right), and 6 months (bottom left) and 4 years (bottom right) after treatment with ALA-PpIX showing 98% tumor clearance.^6^ (b) CT scans of a patient with head and neck cancer before (left) and after (right) Photofrin-PDT, showing only partial tumor destruction as indicated by the white arrows (courtesy Dr Merrill Biel). (c) Reconstruction from MR images of the zones of tissue necrosis (dark blue) following PDT with a vascular-targeted type 1 photosensitizer (WST09) in recurrent prostate cancer (prostate: light blue), using multiple interstitial optical fibers for light delivery. This shows the heterogeneous PDT-induced tissue necrosis within the target volume: note also the off-target damage to the normal rectal wall in the third patient.^7^ (d) Photo during HpD-mediated PDT for recurrent nasopharyngeal cancer in which the tumor showed no treatment response (courtesy Dr. Catherine Tanser).

Despite these advantages, PDT has not yet been widely adopted into routine oncological practice, except for some limited indications and in some countries. One common factor has been the difficulty of achieving reproducible treatment responses across patients and tumor types/stages, as illustrated in Fig. 1. This is due, at least in part, to the fact that, in the majority of approved clinical applications and in many clinical trials to date, the “dosimetry” employed has not advanced substantially beyond the level criticized 40 years ago by Profio and Doiron,^1^ as cited above, despite many developments at the preclinical level.

In general, incomplete tumor destruction is due to one or a combination of: insufficient light energy distribution throughout the tumor, insufficient local photosensitizer concentration and/or suboptimal cellular/subcellular localization, insufficient tissue oxygenation, and/or low intrinsic photodynamic sensitivity at the cell and/or tissue level.

The required accuracy for a particular PDT case depends on several factors, including the specific uptake of the photosensitizer in the tumor relative to surrounding normal tissue and the clinical impact of any off-target damage to the host or adjacent normal tissues. Such damage typically heals within a short period, preserving normal host tissue structure and function. However, in cases where the normal tissue toxicity has a significant clinical impact (e.g., fistula of the hollow tumor-host organs or destruction of eloquent areas of the brain), dosimetry must include the impact on these normal tissues. This requires greater accuracy in the measurement of factors such as light fluence delivery/measurement, photosensitizer tumor uptake and selectivity, tissue oxygenation, and singlet oxygen production, as well as knowledge of the tissues’ intrinsic sensitivities.^8^

## 2 Status of PDT Dosimetry

**Fig. 2:**
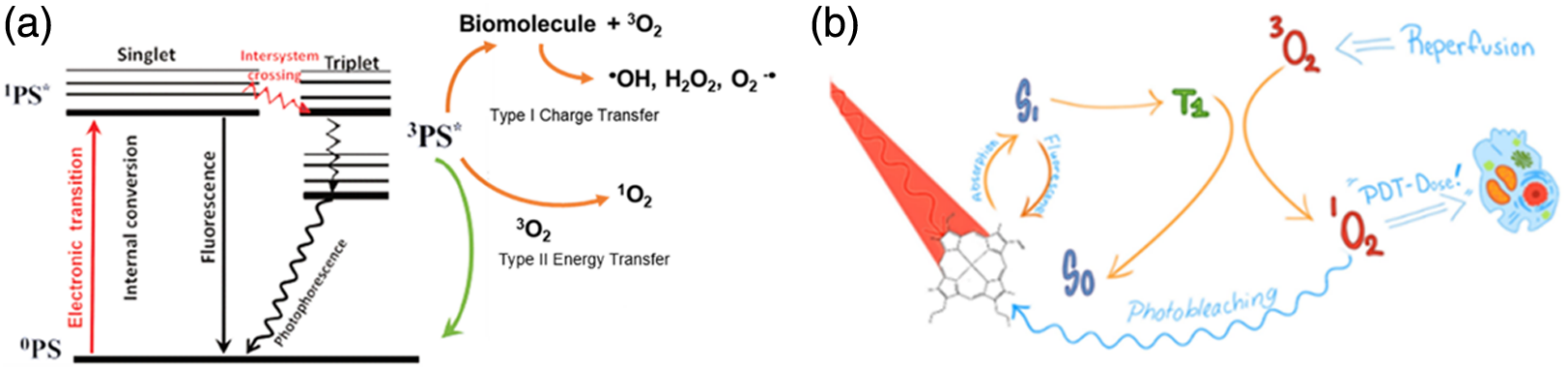
(a) Simplified Jablonski (energy) diagram of PDT photophysics showing absorption of a photon by ground-state photosensitizer, which is excited to a singlet state. This can return to the ground state non-radiatively (generating heat) or by fluorescence emission. Alternatively, the singlet state can undergo intersystem crossing to a long-lived triplet state, which can then interact with triplet ground-state oxygen (O32) via energy transfer, generating cytotoxic singlet oxygen, O12 (type II) or via charge transfer to biomolecules (type I) that result in other oxygen radicals. (b) Corresponding simplified model of PDT processes showing also singlet oxygen–dependent photobleaching.

By the term “PDT dosimetry,” we mean the measurement of physical and biophysical factors involved in the treatment to improve biological or clinical outcomes. To date, it has not included “biological metrics,” as we will discuss below in the context of photochemical immune stimulation. It is important to note that in the following discussion, the use of dosimetry, particularly quantitatively, is much more widespread and advanced in the preclinical research setting than in routine clinical use. One of the major challenges is to achieve clinically practical and cost-effective methods and systems. The main photophysical processes involved in PDT that directly impact biophysical dosimetry are illustrated in Fig. 2.

### 2.1 Singlet Oxygen Dosimetry

**Fig. 3:**
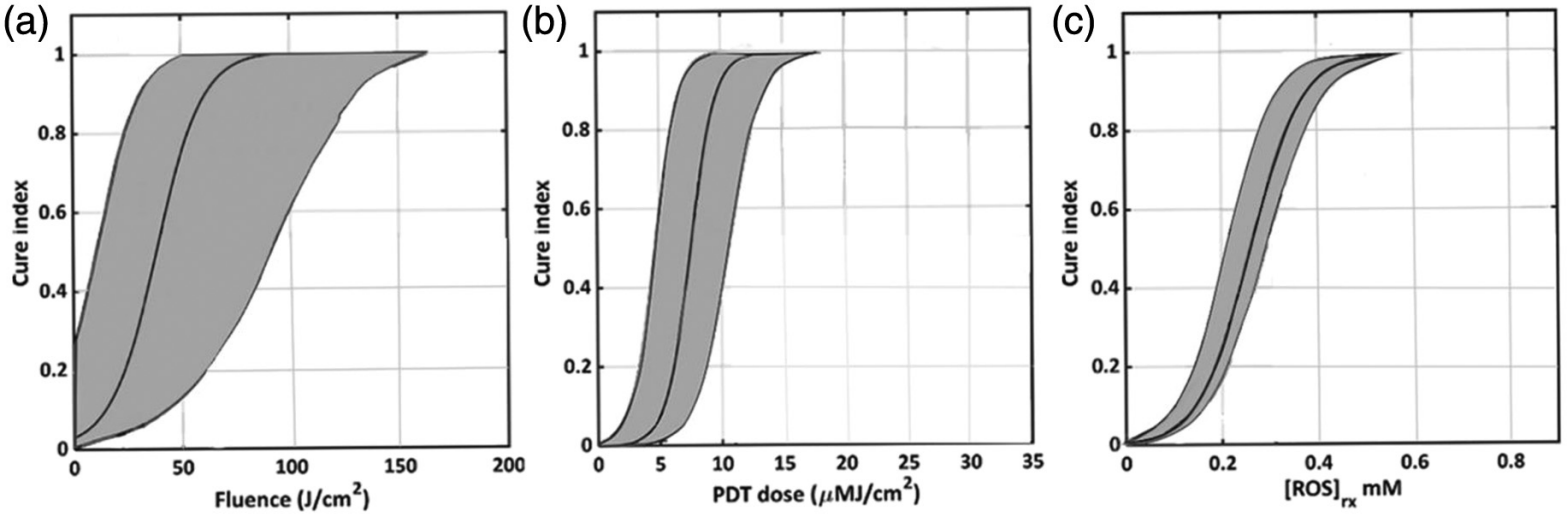
Examples of tumor response (here denoted by “cure index”) in mouse tumor xenografts *in vivo* versus three different PDT dose metrics (after Sheng et al.^10^ with permission): (a) administered light fluence, (b) drug-light product, and (c) concentration of PDT-generated O12 reactive oxygen species. The grey shading indicates the 95% confidence interval for the cure index as function of the different dose metrix.

For type II photosensitization, multiple *in vitro* and *in vivo* studies have demonstrated (with some exceptions^9^) a strong dependence of the tumor response on the volume-averaged or localized cumulative concentration of O12, as illustrated in Fig. 3.

Hence, singlet oxygen luminescence dosimetry (SOLD) based on monitoring the near-infrared (∼1270  nm) light emitted in the O12→O32 transition should be the most powerful approach to PDT dosimetry. However, this is technically very challenging due to the high reactivity of O12 that results in a short (≪1μs) lifetime in cells and tissues,^11^ the relatively high and variable background due to photosensitizer and tissue autofluorescence/phosphorescence, and the low quantum yield of conventional silicon-based photodetectors at this wavelength. Other indirect approaches to PDT dosimetry, as discussed below, also have significant limitations, so that defining and reliably measuring the PDT “dose” remains a significant challenge to clinical translation, especially for non-superficial (i.e., volumetric) treatments with curative intent for which optimal dose delivery is important to achieve the desired clinical outcome.

Having been first reported unequivocally in 1991,^12^ several different SOLD systems have been developed,^13^^–^^18^ including those reported by Yang et al.^19^ in this issue, but they have not yet been adopted clinically due to ongoing technical complexity and cost. As a rule, it is desirable to use time-resolved detection with a time gate to eliminate the tissue and photosensitizer background fluorescence and phosphorescence signals. The use of novel single-photon detectors with high sensitivity and ns pulsed diode lasers PDT-treatment wavelengths is being explored by several groups toward instruments that are clinically practical. The majority of SOLD studies are based on point-by-point measurements using fiber-optic probes. Direct luminescence imaging is also possible, as demonstrated by Lin et al.^20^ who imaged O12 generation in blood vessels during PDT of port-wine stains. However, in this case, the photosensitizer and oxygen concentrations within the vessels were very high and generated strong luminescence signals that facilitated direct imaging. The applicability to tumor PDT will be more challenging.

An alternative strategy, “indirect O12 dosimetry,” is based on combinations of measured or estimated/assumed local light fluence, photosensitizer concentration, and tissue oxygenation that can be combined in a biophysical model to calculate the O12 concentration in the target tissue^21^ (see below). Two additional indirect approaches have been used in preclinical research. The first is the use of fluorescence “reporters,” most commonly singlet oxygen sensor green (SOSG), which becomes fluorescent upon interaction with O12 and so can be used to measure or image its distribution.^22^ This has proved very useful in preclinical models, as in Yang et al.,^23^ for example, who demonstrated that organosilica coated with prussian blue was an effective oxygen delivery nanoplatform with high PDT efficacy. The second, very recent approach is to use a thrombin-activated molecular beacon composed of pyropheophorbide-a as photosensitizer and 5-carboxy-X-rhodamine as fluorophore, which relies on the conversion of prothrombin into thrombin by O12 and subsequent fluorescence emission from the rhodamine.^24^

### 2.2 Explicit Dosimetry

The concept here is to measure the separate factors that produce an effective PDT treatment: the spatially resolved light fluence and, in some cases, fluence rate; the volume-averaged photosensitizer concentration in the target and adjacent tissues; and the local or volume-averaged tissue oxygenation. Various integrated systems have been developed for this approach, with the most comprehensive to date utilizing multiple interstitial optical fibers for both light delivery and the measurement of one or more of these factors. The measurements are combined into an effective dose using a biophysical model (e.g., assuming a PDT dose threshold). This procedure may also be combined with pre-treatment planning to determine the best placement and form of the fibers (e.g., isotropic point or cylindrical diffusing tips) and deliver light energy to each fiber.^25^ Benefits have been demonstrated in clinical trials,^26^ and at least one system^27^ is being commercialized. Although other companies are working on alternative treatment planning approaches, such as SimphoSOFT (see Ref. 28) or Modulight Cloud analytics,^29^ no routine clinical use of these explicit dosimetry approaches is known. Various academic groups are also working on explicit dosimetry approaches with online monitoring.^30^^–^^50^

Specifically with respect to determining light distributions in tissue, we note that many of the early developments in the modeling of light propagation in tissue (Monte Carlo computer modeling^51^ and diffusion theory^52^) and in experimental techniques^53^ for measuring tissue absorption and scattering properties were motivated by PDT dosimetry, and these have since been adopted across a wide range of biophotonics applications. An example of direct light fluence/rate measurements in patients is real-time monitoring during PDT of large and complex body cavity surfaces (peritoneal, thoracic) using calibrated photodetectors placed at multiple points on the irradiated tissue surface. This has been used in clinical trials to ensure the best uniformity of light delivery by dynamic adjustment of the light source placement based on these real-time measurements.^33^ A second example of direct light fluence monitoring is in whole bladder-wall treatments using isotropic fiber-optic probes (originally developed by Star and colleagues^54^) to account for the additional “backscattered” light that depends on the diffuse reflectivity of the tissue.^55^ Indeed, following the global-first government approval for PDT (1993) to treat recurrent superficial bladder cancer, this highly effective treatment was never adopted into clinical use because of post-treatment complications due to overexposure of the normal bladder wall. This application, with appropriate light dosimetry, is being revisited in current clinical trials.^39^^,^^56^

Photosensitizer “dose” (concentration in tissue) measurements have been implemented less commonly in patients, although techniques and devices suitable for this purpose have been developed,^57^^,^^58^ primarily based on photosensitizer fluorescence point spectroscopy or wide-field imaging, in combination with diffuse reflectance measurements to correct for variable light attenuation in tissue.^59^^–^^61^ (We note that fluorescence imaging in PDT contributed significantly to the development of fluorescence-guided tumor surgery, including quantitative techniques.^62^^–^^64^) Analogous to chemotherapy drugs, a fixed amount of photosensitizer per kg body weight or surface area is usually administered, based on an “optimum” found in dose escalation/de-escalation clinical trials, balancing efficacy against toxicities. However, this ignores the often marked patient-to-patient and tumor-to-tumor variations in photosensitizer uptake and likely contributes significantly to the variability in responses.

For type-II photosensitizers, the cell/tissue oxygenation is the third main component in O12 generation. This is seldom reported in either animal models or patients, with it usually being assumed that oxygen is available in excess, despite the known hypoxic nature of many tumors.^65^ The ${\mathrm{pO}}_{2}$ may be measured directly using interstitial needle probes based on Clark electrodes or optical interactions, and this has been used in some preclinical studies.^66^^,^^67^ As a surrogate, the hemoglobin saturation, ${\mathrm{SO}}_{2}$, may be determined by diffuse optical spectroscopy/imaging based on the differences in the absorption spectra between ${\mathrm{HbO}}_{2}$ and Hb. Point ${\mathrm{SO}}_{2}$ measurements have been incorporated into some explicit dosimetry instruments and can be used in conjunction with light and photosensitizer measurements to generate a more accurate estimate of the PDT effective dose, without assuming unlimited oxygen.^68^ Most recently, in the specific case of (ALA-)PpIX, Pogue and colleagues^38^^,^^69^^,^^70^ have used electron paramagnetic resonance (EPR) oximetry to measure the tissue oxygenation, which could be incorporated into PDT dosimetry for this particular photosensitizer.

### 2.3 Implicit Dosimetry

Photobleaching of photosensitizer, as reported by a drop in fluorescence intensity [see Fig. 2(b)], has been used preclinically^69^^,^^70^ and in limited clinical studies in the head and neck^70^ and skin tumors^38^ as a surrogate measure of the effective PDT dose delivered. The underlying assumption is that photobleaching is caused by the molecular alteration of the photosensitizer by singlet oxygen. The drop in fluorescence can be monitored using either a fiberoptic probe or fluorescence imaging (including intraoperative and endoscopic applications). For example, Cottrell et al. imaged the fluorescence from ALA-PpIX in skin tumors during PDT and showed that 80% of PpIX’s fluorescence bleaching was achieved in 2.5 to 3.5 min in the 10 to $150\;\mathrm{mW}\cdot {\mathrm{cm}}^{-2}$ range.^71^ A limitation of photobleaching-based implicit dosimetry is that the interpretation of measurements can be confounded by multiple factors, including photosensitizer destruction/modification that is not mediated by O12. Nevertheless, Siddiqui et al.^72^ showed that >40% PpIX fluorescence reduction post PDT for oral cancer, as measured with a low-cost imaging system, was indicative of a successful therapeutic session.

### 2.4 PDT Threshold Model

A common observation in preclinical and clinical PDT studies is the existence of a distinct boundary between tissue destruction (necrosis) and no significant effect. Farrell et al.^73^ initially proposed that this can be interpreted as meaning that PDT shows a “threshold” behavior, i.e., a certain minimum cumulative O12 level has to be generated per unit tissue volume to cause tissue necrosis. This contrasts with the response to ionizing radiation that is stochastic, i.e., varies continuously and probabilistically with dose, so that there is a continuous gradient of tissue damage with depth. (This can also be the case in PDT mediated by cell apoptosis.^74^) The threshold behavior can be incorporated into a PDT dose model to enable systematic and quantitative optimization of treatment delivery in, e.g., multifiber irradiation of prostate cancer, as in Fig. 1(c).^7^ In this case, by fitting the necrosis boundary (determined by MRI post-treatment) from the combined fiber irradiations to the calculated light dose distribution (based on diffusion theory or MC simulations and assuming uniform photosensitizer distribution), the T values could be determined in each patient. The running average of the T values across patients can then be used to plan subsequent treatments.

### 2.5 Summary of Current PDT Dosimetry

Table 1 summarizes the various dosimetry approaches discussed above, along with their roles in different clinical scenarios and the limitations and challenges associated with these uses. As discussed by Komolibus et al.,^41^ PDT dosimetry by itself does not present a complete framework for how to optimize PDT treatments. In addition, the impact of fluence rate, particularly in relation to the deoxygenation of the target tissue, is often overlooked.^75^

**Table 1:** PDT dosimetry approaches and their applicability to different clinical situations.

Approach	Appropriate clinical situation		Limitations/challenges
Fixed administered prescription (PS, DLI, irradiance)	Class I	• Simple accessible target geometry	• Uncontrolled variances
		• Continuum of useful efficacy	
		• High target/non-target PS uptake	• No “personalization”
Measurement of PS photobleaching	Class II	• Surface (incl. endoscopic) to minimize “missed” areas.	• Only for photolabile PS
		• Photobleaching represents the volumetric cytotoxic dose equivalent.	• Uncertain reliability
Fixed *in situ* prescription	Class III	• Curative intent for localized volumetric tumors	• Technical uncertainty in measurements of light and [PS]
Indirect O12 measurements		• Interstitial delivery	
		• Possible surrounding normal structures that limit dose delivery	• Assumed threshold response, but currently no measured outcomes
			• Assumed homogeneity may mask true response
Singlet oxygen luminescence	Class IV	• Curative intent for localized volumetric tumors	• Cost/complexity of use
Light-photosensitizer-oxygen measurements		• Interstitial delivery	• Not ready for routine clinical use
		• Possible surrounding normal structures that limit dose delivery	• Tissue response/outcome database

Pre-treatment planning can be used to optimize light source placements and their power delivery, particularly for interstitial applications. Such planning usually accounts for some or all of these factors: (i) expected photosensitizer uptake in the target and surrounding tissues; (ii) the sensitivities of these tissues to PDT; and (iii) oxygen reperfusion. Planning systems have been presented for particular indications, such as pleural cancer PDT,^76^ prostate cancer,^7^^,^^77^ gliomas,^43^ and central airway tumors,^32^ as well as indication-independent platforms.^36^^,^^78^^,^^79^

### 2.6 Comparison with Radiotherapy Dosimetry

Finally, comparisons may be drawn^80^ between PDT dosimetry and dosimetry in radiation therapy (Rx), with which it shares the use of controlled EM energy to destroy target (tumor) tissue while minimizing off-target damage. Physical dosimetry in Rx is much more precise than in PDT and is used universally to ensure delivery of a safe and effective dose within tight tolerances. In contrast, PDT dosimetry is more challenging because

• the optical properties of tissue that determine the spatial distribution of light are much more variable from tumor to tumor and heterogeneous within any given tumor than are the absorption and (relatively much lower) scattering properties at therapeutic X-ray energies, where soft tissues are largely “water equivalent”;
• these tissue properties are also strongly wavelength dependent;
• can change dynamically during treatment
• the absorbed light dose–depth gradients in PDT are very large, making the desired light dose more difficult to achieve across the tumor target;
• PDT has the added factor of photosensitizer uptake and heterogeneous intratumoral distribution;
• PDT is usually delivered as a single treatment, whereas fractionated dose delivery is common in Rx, allowing “adaptive” corrections to be made between fractions to account for the actual delivered dose and changes in anatomy;
• the biological responses are also very different, with Rx largely exploiting proliferative cell death through DNA damage, rather than the complex multiple cell-death pathways in PDT that make it difficult to show a one-to-one correspondence between physical dose and biological outcome. Additionally, a long history of detailed, multicenter clinical studies has generated data on tissue responses to radiation that are used to define prescription doses for various types of tumors (see, for example,^81^) and dose limits for normal tissue structures.^82^^,^^83^

## 3 PDT Immune Effects: Impact on Dosimetry

**Fig. 4:**
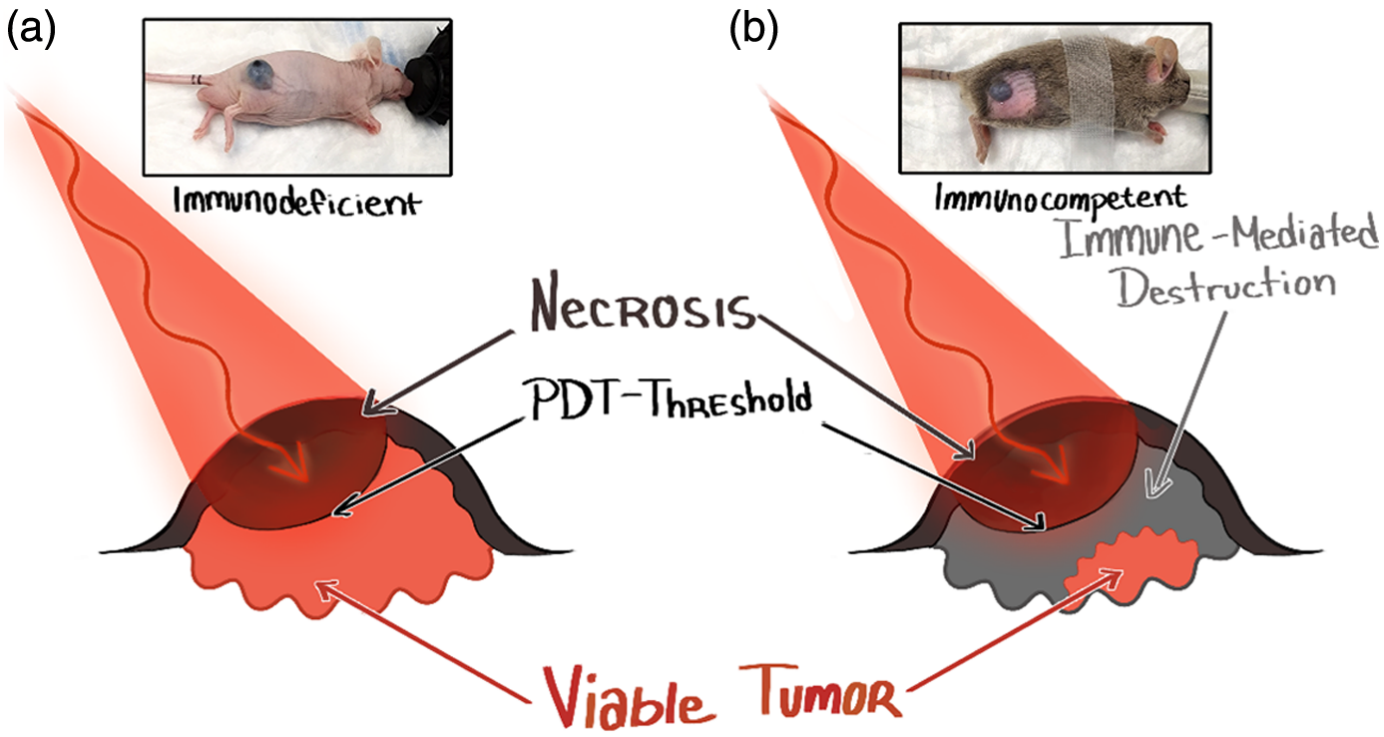
Schematic of PDT/PCIST treatment of pigmented melanoma in (a) immunodeficient and (b) immunocompetent animals (after Pires et al.^86^).

The above approaches to PDT dosimetry are based on the target tumor tissue being destroyed by the photoproduct (e.g., O12) of light-activated photosensitizer, either by direct tumor cell kill and/or by destruction of the tumor microvasculature. That is, there is a direct relationship between delivered biophysical dose and the (acute) response of the treated tumor (e.g., none, partial, and complete). However, the secondary effect of PDT on the host immune system is increasingly recognized as playing an important role, both in eradicating the primary treated tumor and potentially in limiting tumor progression and metastasis.^84^^,^^85^ Thereby, the tumor response becomes “uncoupled” from the biophysical PDT dose. An example, based on recent preclinical findings by Pires et al.,^86^ of PDT treatment of cutaneous pigmented melanoma is illustrated in Fig. 4. In mice lacking an immune system, tumors up to ∼1  mm thick could be eradicated (but with no impact on survival), whereas in immunocompetent mice receiving exactly the same treatment, i.e., delivery of the same biophysical dose to the tumor, tumors at least 4 mm thick were destroyed. Since the light attenuation in pigmented melanoma is very high due to the melanin absorption, a negligible light dose is delivered beyond about 1 mm depth. As confirmed by functional and mechanistic assays, the deeper-lying tumor tissue was destroyed by recruitment and activation of cytotoxic immune cells in response to the inflammatory processes initiated by the “direct” PDT damage to the more superficial tumor. This has been referred to as PhotoChemical Immune Stimulation Therapy (PCIST).

Different conceptual models of the effective PCIST dose could be considered; thus, the total effect on the primary treated tumor may be described by

$$D_{\text{PCIST-primary}}=D_{\text{PDT-primary}}x\{1+\mathrm{ISF}\},$$ (1a)

or

$$D_{\mathrm{PC}\text{I}\mathrm{ST}\text{-primary}}=D_{\text{PDT-primary}}x\{1+\mathrm{ISF}(D_{\text{PDT-primary}})\},$$ (1b)

where $D_{\text{PDT-primary}}$ is the biophysical dose to the primary tumor, and ISF is a measure of the strength of the immune stimulation. Equation (1a) implies that the immune response essentially “amplifies” the biophysical dose. Equation (1b) assumes further that the strength of the immune response is itself a function of the biophysical dose, as indicated by the term $\mathrm{ISF}(D_{\text{PDT-primary}})$, a possible mechanism being that the cell-death pathways, which have different immune-stimulation effects, are dose-dependent. The above addresses only the PCIST response to primary disease and does not address the important aspect of this dose on metastatic disease.

How, then, could a “PCIST dose” be defined and measured such that it would correlate with—or, ideally—predict the full-thickness tumor destruction and/or modification of metastatic tumor? This is far from straightforward, and development remains at an early stage. Possible approaches, some of which may not easily be clinically translated, include:

(1) performing immune profiling prior to treatment to estimate, based on retrospective analysis of data from previous patients (or animals in the case of preclinical studies), the likely strength of the immune response and its impact on treatment outcomes,
(2) monitoring the anti-tumor immune response following treatment, such as by quantitative imaging of infiltrating cytotoxic immune cells into the tumor using endogenous or exogenous fluorescence^87^ or optically active nanoparticles (e.g., surface enhanced Raman scattering, SERS^88^) to correlate with the outcome and incorporate this information into subsequent PCIST treatment planning;
(3) monitoring the overall tumor response by tracking blood biomarkers, including cytokines and chemokines as early markers, and circulating tumor cells, cell-free DNA, or extracellular vesicles as later markers;
(4) measuring immune biomarkers after a sub-therapeutic “test” dose to inform the subsequent full-treatment dose.

Other fundamental questions about the PCIST response that will affect how effective doses might be defined and measured include:

(a) Is there a minimum biophysical dose and/or minimum mass of tissue (relative to the total body mass) receiving at least the PDT threshold dose that is required to generate an effective immune response of the primary tumor?
(b) Does the immune response increase monotonically with the biophysical dose, or is there an optimum dose, as suggested in recent studies^89^ using porphyrin–lipid nanoparticles as the photosensitizer, where the response versus administered light fluence showed a peak (at $25\;J\;{\mathrm{cm}}^{-2}$, which is much lower than typical PDT light doses)?
(c) How does ISF depend on the cell-death pathways involved in the primary treatment, recognizing that the relative contributions of the different pathways will vary with depth in the tumor as the biophysical PDT dose decreases?
(d) What is the optimum treatment protocol to generate a significant PCIST response?
(e) Can the probability of systemic immune impact on either the risk of tumor progression and metastatic spread or on existing metastatic tumor be predicted from some measure of PCIST dose?

## 4 Conclusion

Many tools have been developed to enable PDT dosimetry and have been used in preclinical research with animal models, thereby contributing to the understanding of the main determinants of the biological efficacy of PDT and resulting outcomes. Over the past four decades, oncological PDT has had many clinical starts and stops, and most clinical practice remains rooted in simply delivering a particular light energy fluence and administered photosensitizer amount at a particular photosensitizer-light interval, with no adjustments from patient to patient other than to match the light delivery geometrically to the target tumor. Thus, the age of more complete and sophisticated PDT dosimetry envisaged by Doiron and Profio above has NOT emerged into widespread clinical practice: even in phase II/III clinical trials, the “effective” dose parameters have largely not been optimized or individualized. This has surely contributed to the large spread in clinical outcomes, which is an impediment to adoption and perhaps to the failure of some of these trials.

PDT is also still evolving in fundamentally different ways that pose additional and largely unexplored dosimetry challenges beyond the conventional techniques. First, as discussed above, it has potential through immune stimulation to extend beyond being a purely local treatment to one with systemic (e.g., anti-metastatic) effects. This will require integration of biological metrics into the definition of PDT “dose”. Second, there are several emerging variants of PDT, including the use of ionizing radiation (radiodynamic therapy, RDT)^90^^–^^93^ or ultrasound (sonodynamic therapy, SDT^94^^,^^95^), rather than light as the energy source for photosensitizer activation. In principle, these approaches will enable treatment of larger and deep-seated tumors while preserving the biological advantages of PDT. Existing X-ray and ultrasound dosimetry techniques will then need to be integrated with components of PDT dosimetry, such as measuring the photosensitizer concentration or the singlet oxygen. An interesting challenge is to determine whether significant immune stimulation can also be achieved with these modalities, because the cell-death pathways that impact immune responses are likely different from those in PDT. Thus, both the “beyond local” and “beyond light” envisaged in these methods will require new approaches to dosimetry and will require close collaborations between the “physics” and “biology” communities. These challenges and opportunities represent an exciting new chapter in the evolution of PDT dosimetry.

## Data Availability

Is not applicable for this perspective paper.

## References

[1] Profio A. E. Doiron D. R., “Dosimetry considerations in phototherapy,” Med. Phys. 8(2), 190–196 (1981). MPHYA6 0094-2405 10.1118/1.594932 7322046

[2] Cuadrado C. F. et al., “Clinical and pre-clinical advances in the PDT/PTT strategy for diagnosis and treatment of cancer,” Photodiagn.s Photodyn. Ther. 50, 104387 (2024). 10.1016/j.pdpdt.2024.104387 39490802

[3] Penetra M. Arnaut L. G. Gomes-da-Silva L. C., “Trial watch: an update of clinical advances in photodynamic therapy and its immunoadjuvant properties for cancer treatment,” OncoImmunology 12(1), 2226535 (2023). 10.1080/2162402X.2023.2226535 37346450 PMC10281486

[4] Moan J. Peng Q., “An outline of the hundred-year history of PDT,” Anticancer Res. 23(5A), 3591–3600 (2003). 14666654

[5] Aebisher D. Szpara J. Bartusik-Aebisher D., “Advances in medicine: photodynamic therapy,” Int. J. Mol. Sci. 25(15), 8258 (2024).1422-0067 10.3390/ijms25158258 39125828 PMC11311490

[6] Oseroff A. R. et al., “Treatment of diffuse basal cell carcinomas and basaloid follicular hamartomas in nevoid basal cell carcinoma syndrome by wide-area 5-aminolevulinic acid photodynamic therapy,” Arch. Dermatol. 141(1), 60–67 (2005). 10.1001/archderm.141.1.60 15655143

[7] Davidson S. R. H. et al., “Treatment planning and dose analysis for interstitial photodynamic therapy of prostate cancer,” Phys. Med. Biol. 54(8), 2293–2313 (2009). PHMBA7 0031-9155 10.1088/0031-9155/54/8/003 19305043

[8] Lilge L. Wilson B. C., “Photodynamic therapy of intracranial tissues: a preclinical comparative study of four different photosensitizers,” J. Clin. Laser Med. Surg. 16(2), 81–91 (1998). JCLSEO 10.1089/clm.1998.16.81 9663099

[9] Wang K. K.-H. Mitra S. Foster T. H., “Photodynamic dose does not correlate with long-term tumor response to mTHPC-PDT performed at several drug-light intervals: photodynamic dose and tumor response to mTHPC-PDT,” Med. Phys. 35(8), 3518–3526 (2008). MPHYA6 0094-2405 10.1118/1.2952360 18777912 PMC2562246

[10] Sheng T. et al., “Reactive oxygen species explicit dosimetry to predict tumor growth for benzoporphyrin derivative-mediated vascular photodynamic therapy,” J. Biomed. Opt. 25(06), 063805 (2020). JBOPFO 1083-3668 10.1117/1.JBO.25.6.063805 31912689 PMC6952881

[11] Niedre M. Patterson M. S. Wilson B. C., “Direct near-infrared luminescence detection of singlet oxygen generated by photodynamic therapy in cells in vitro and tissues *in vivo*,” Photochem. Photobiol. 75(4), 382–391 (2002). PHCBAP 0031-8655 10.1562/0031-8655(2002)0750382DNILDO2.0.CO2 12003128

[12] Tromberg B. J. Dvornikov T. B. Berns M. W., “Indirect spectroscopic detection of singlet oxygen during photodynamic therapy,” Proc. SPIE 1427, 101–108 (1991). PSISDG 0277-786X 10.1117/12.44091

[13] Jarvi M. T. et al., “Singlet Oxygen Luminescence Dosimetry (SOLD) for photodynamic therapy: current status, challenges and future prospects,” Photochem. Photobiol. 82(5), 1198–1210 (2006). PHCBAP 0031-8655 10.1562/2006-05-03-IR-891 16808593

[r14] Hackbarth S. et al., “Singlet oxygen phosphorescence detection *in vivo* identifies PDT-induced anoxia in solid tumors,” Photochem. Photobiol. Sci. 18(6), 1304–1314 (2019). PPSHCB 1474-905X 10.1039/c8pp00570b 30994640

[r15] Kim I.-W. et al., “Direct measurement of singlet oxygen by using a photomultiplier tube-based detection system,” J. Photochem. Photobiol. B 159, 14–23 (2016). JPPBEG 1011-1344 10.1016/j.jphotobiol.2016.03.012 26995671

[r16] Kim M. et al., “A comparison of Singlet Oxygen Explicit Dosimetry (SOED) and Singlet Oxygen Luminescence Dosimetry (SOLD) for photofrin-mediated photodynamic therapy,” Cancers 8(12), 109 (2016). 10.3390/cancers8120109 27929427 PMC5187507

[r17] Ong Y. H. et al., “Reactive oxygen species explicit dosimetry for photofrin-mediated pleural photodynamic therapy,” Photochem. Photobiol. 96(2), 340–348 (2020). PHCBAP 0031-8655 10.1111/php.13176 31729774 PMC7299188

[18] Boso G. et al., “Time-Resolved singlet-oxygen luminescence detection with an efficient and practical semiconductor single-photon detector,” Biomed. Opt. Express 7(1), 211 (2016). BOEICL 2156-7085 10.1364/BOE.7.000211 26819830 PMC4722905

[19] Yang W. et al., “Comparison of Multispectral Singlet Oxygen Luminescence Dosimetry (MSOLD) and Singlet Oxygen Explicit Dosimetry (SOED) in in-vitro BPD-Mediated Photodynamic Therapy (PDT),” J. Biomed. Opt, this issue.

[20] Lin L. et al., “Singlet oxygen luminescence image in blood vessels during vascular-targeted photodynamic therapy,” Photochem. Photobiol. 96(3), 646–651 (2020). PHCBAP 0031-8655 10.1111/php.13264 32220067

[21] Vikas V. et al., “Analysis of singlet oxygen luminescence generated by protoporphyrin IX,” Antioxidants 14(2), 176 (2025). 10.3390/antiox14020176 40002363 PMC11851838

[22] Kim S. Fujitsuka M. Majima T., “Photochemistry of singlet oxygen sensor green,” J. Phys. Chem. B 117(45), 13985–13992 (2013). JPCBFK 1520-6106 10.1021/jp406638g 24111566

[23] Yang Z. L. et al., “Oxygen-evolving mesoporous organosilica coated Prussian blue nanoplatform for highly efficient photodynamic therapy of tumors,” Adv. Sci. 5(5), 1700847 (2018). 10.1002/advs.201700847 PMC5980201 29876209

[24] Lin H. et al., “Simultaneous direct and indirect assessments of singlet oxygen generation during vascular-targeted photodynamic therapy with thrombin molecular beacon,” Photochem. Photobiol. 101, 1307–1316 (2025). PHCBAP 0031-8655 10.1111/php.14118 40405469

[25] Johansson A. et al., “Realtime light dosimetry software tools for interstitial photodynamic therapy of the human prostate,” Med. Phys. 34(11), 4309–4321 (2007). MPHYA6 0094-2405 10.1118/1.2790585 18072496

[26] Swartling J. et al., “System for interstitial photodynamic therapy with online dosimetry: first clinical experiences of prostate cancer,” J. Biomed. Opt. 15(5), 058003 (2010). JBOPFO 1083-3668 10.1117/1.3495720 21054129

[27] https://spectracure.com/en/ (accessed 4 December 2025).

[28] https://simphotek.net/app-pdt.html (accessed 4 December 2025).

[29] https://cloud.modulight.com/ (accessed 4 December 2025).

[30] Wang X. et al., “Three-dimensional image-guided topical photodynamic therapy system with light dosimetry dynamic planning and monitoring,” Biomed. Opt. Express 14(1), 453 (2023). BOEICL 2156-7085 10.1364/BOE.481248 36698654 PMC9842015

[r31] Wang S. et al., “Integrating clinical access limitations into iPDT treatment planning with PDT-SPACE,” Biomed. Opt. Express 14(2), 714 (2023). BOEICL 2156-7085 10.1364/BOE.478217 36874501 PMC9979674

[r32] Oakley E. et al., “Computational optimization of irradiance and fluence for interstitial photodynamic therapy treatment of patients with malignant central airway obstruction,” Cancers 15(9), 2636 (2023). 10.3390/cancers15092636 37174102 PMC10177073

[r33] Sun H. et al., “Clinical PDT dose dosimetry for pleural photofrin-mediated photodynamic therapy,” J. Biomed. Opt. 29(01), 018001 (2024). JBOPFO 1083-3668 10.1117/1.JBO.29.1.018001 38223299 PMC10787190

[r34] Finlayson L. et al., “Simulating photodynamic therapy for the treatment of glioblastoma using Monte Carlo radiative transport,” J. Biomed. Opt. 29(02), 025001 (2024). JBOPFO 1083-3668 10.1117/1.JBO.29.2.025001 38322729 PMC10846422

[r35] Jacques S. L., “The optical dosimetry of PDT,” Proc. SPIE 13296, 1329602 (2025). 10.1117/12.3046144

[r36] Chamberlain S. et al., “Image-based treatment planning for TLD1433 mediated intraoperative photodynamic therapy with an optical surface applicator—a translational rodent study,” Photochem. Photobiol. 101(5), 1279–1290 (2025). PHCBAP 0031-8655 10.1111/php.14101 40357896 PMC12353992

[r37] Greer A., “*In vivo* tissue evaluation reveals improvements in explicit PDT dosimetry,” Photochem. Photobiol. 96(2), 437–439 (2020). PHCBAP 0031-8655 10.1111/php.13225 32060926

[r38] Ruiz A. J. et al., “Smartphone fluorescence imager for quantitative dosimetry of protoporphyrin-IX-based photodynamic therapy in skin,” J. Biomed. Opt. 25(06), 063802 (2019). JBOPFO 1083-3668 10.1117/1.JBO.25.6.063802 31820594 PMC6901011

[r39] Lilge L. et al., “Minimal required PDT light dosimetry for nonmuscle invasive bladder cancer,” J. Biomed. Opt. 25(06), 068001 (2020). JBOPFO 1083-3668 10.1117/1.JBO.25.6.068001 32529817 PMC7289452

[r40] McLellan L. J. et al., “SmartPDT®: smartphone enabled real-time dosimetry via satellite observation for daylight photodynamic therapy,” Photodiagn. Photodyn. Ther. 31, 101914 (2020). 10.1016/j.pdpdt.2020.101914 PMC7336930 32645436

[r41] Komolibus K. et al., “Perspectives on interstitial photodynamic therapy for malignant tumors,” J. Biomed. Opt. 26(07), 070604 (2021). JBOPFO 1083-3668 10.1117/1.JBO.26.7.070604 34302323 PMC8299827

[r42] Beigzadeh A. M. et al., “A new optical method for online monitoring of the light dose and dose profile in photodynamic therapy,” Lasers Surg. Med. 52(7), 659–670 (2020). LSMEDI 0196-8092 10.1002/lsm.23193 31777113

[r43] Aumiller M. et al., “Individualization of interstitial photodynamic therapy for malignant gliomas,” Proc. SPIE 11079, 110790P (2019). PSISDG 0277-786X 10.1117/12.2527115

[r44] Gregor A. Sase S. Wagnieres G., “Optimization of the distance between cylindrical light distributors used for interstitial light delivery in biological tissues,” Photonics 9(9), 597 (2022). 10.3390/photonics9090597

[r45] Moritz T. J. et al., “Multispectral singlet oxygen and photosensitizer luminescence dosimeter for continuous photodynamic therapy dose assessment during treatment,” J. Biomed. Opt. 25(6), 063810 (2020). JBOPFO 1083-3668 10.1117/1.JBO.25.6.063810 32170859 PMC7068220

[r46] Garcia M. R. et al., “Development of a system to treat and online monitor photodynamic therapy of skin cancer using PpIX near-infrared fluorescence,” Photodiagn. Photodyn. Ther. 30, 101680 (2020). 10.1016/j.pdpdt.2020.101680 32006649

[r47] Spring B. Q. et al., “Illuminating the numbers: integrating mathematical models to optimize photomedicine dosimetry and combination therapies,” Front. Phys. 7, 46 (2019). 10.3389/fphy.2019.00046 31123672 PMC6529192

[r48] Dupont C. et al., “Photodynamic therapy for glioblastoma: a preliminary approach for practical application of light propagation models,” Lasers Surg. Med. 50(5), 523–534 (2018). LSMEDI 0196-8092 10.1002/lsm.22739 28906571

[r49] Leroy H.-A. et al., “Interstitial photodynamic therapy for glioblastomas: a standardized procedure for clinical use,” Cancers 13(22), 5754 (2021). 10.3390/cancers13225754 34830908 PMC8616201

[50] Betrouni N. et al., “Real-time light dosimetry for intra-cavity photodynamic therapy: application for pleural mesothelioma treatment,” Photodiagn. Photodyn. Ther. 18, 155–161 (2017). 10.1016/j.pdpdt.2017.02.011 28254624

[51] Wilson B. C. Adam G., “A Monte Carlo model for the absorption and flux distributions of light in tissue,” Med. Phys. 10(6), 824–830 (1983). MPHYA6 0094-2405 10.1118/1.595361 6656695

[52] Yoon G. Prahl S. A. Welch A. J., “Accuracies of the diffusion approximation and its similarity relations for laser irradiated biological media,” Appl. Opt. 28(12), 2250 (1989). APOPAI 0003-6935 10.1364/AO.28.002250 20555507

[53] Wilson B. C. Patterson M. S., “The physics of photodynamic therapy,” Phys. Med. Biol. 31(4), 327–360 (1986). PHMBA7 0031-9155 10.1088/0031-9155/31/4/001 3526361

[54] Star W. M., “Light dosimetry *in vivo*,” Phys. Med. Biol. 42(5), 763–787 (1997). PHMBA7 0031-9155 10.1088/0031-9155/42/5/003 9172258

[55] Staveren H. J. V. et al., “Integrating sphere effect in whole bladder wall photodynamic therapy. I. 532 Nm versus 630 Nm optical irradiation,” Phys. Med. Biol. 39(6), 947–959 (1994). PHMBA7 0031-9155 10.1088/0031-9155/39/6/003 15551572

[56] Kulkarni G. S. et al., “A phase 1b clinical study of intravesical photodynamic therapy in patients with bacillus Calmette-Guérin–unresponsive non–muscle-invasive bladder cancer,” Eur. Urol. Open Sci. 41, 105–111 (2022). 10.1016/j.euros.2022.04.015 35813250 PMC9257636

[57] Zhou X. et al., “Pretreatment photosensitizer dosimetry reduces variation in tumor response,” Int. J. Radiat. Oncol. 64(4), 1211–1220 (2006). 10.1016/j.ijrobp.2005.11.019 16504761

[58] Saeidi T. et al., “Photosensitizer spatial heterogeneity and its impact on personalized interstitial photodynamic therapy treatment planning,” J. Biomed. Opt. 30(1), 018001 (2025). JBOPFO 1083-3668 10.1117/1.JBO.30.1.018001 39802351 PMC11724368

[59] Weersink R. A. et al., “Accuracy of noninvasive in vivo measurements of photosensitizer uptake based on a diffusion model of reflectance spectroscopy,” Photochem. Photobiol. 66(3), 326–335 (1997). PHCBAP 0031-8655 10.1111/j.1751-1097.1997.tb03155.x 9297977

[r60] Kim A. et al., “Quantification of in vivo fluorescence decoupled from the effects of tissue optical properties using fiber-optic spectroscopy measurements,” J. Biomed. Opt. 15(6), 067006 (2010). JBOPFO 1083-3668 10.1117/1.3523616 21198210 PMC3025598

[61] Amelink A. et al., “Monitoring PDT by means of superficial reflectance spectroscopy,” J. Photochem. Photobiol. B 79(3), 243–251 (2005). JPPBEG 1011-1344 10.1016/j.jphotobiol.2005.01.006 15896651

[62] Hoda M. R. Popken G., “Surgical outcomes of fluorescence-guided laparoscopic partial nephrectomy using 5-aminolevulinic acid-induced protoporphyrin IX,” J. Surg. Res. 154(2), 220–225 (2009). JSGRA2 0022-4804 10.1016/j.jss.2008.12.027 19375717

[r63] Stummer W. et al., “Fluorescence-guided surgery with 5-aminolevulinic acid for resection of malignant glioma: a randomised controlled multicentre phase III trial,” Lancet Oncol. 7(5), 392–401 (2006). LOANBN 1470-2045 10.1016/S1470-2045(06)70665-9 16648043

[64] Inoue K. et al., “Comparison between intravesical and oral administration of 5-aminolevulinic acid in the clinical benefit of photodynamic diagnosis for nonmuscle invasive bladder cancer,” Cancer 118(4), 1062–1074 (2012). CANCAR 0008-543X 10.1002/cncr.26378 21773973

[65] Bhandari V. et al., “Molecular landmarks of tumor hypoxia across cancer types,” Nat. Genet. 51(2), 308–318 (2019). NGENEC 1061-4036 10.1038/s41588-018-0318-2 30643250

[66] Piffaretti F. et al., “Real-time, *in vivo* measurement of tissular pO2 through the delayed fluorescence of endogenous protoporphyrin IX during photodynamic therapy,” J. Biomed. Opt. 17(11), 115007 (2012). JBOPFO 1083-3668 10.1117/1.JBO.17.11.115007 23214178

[67] Pogue B. W. et al., “Analysis of the heterogeneity of pO2 dynamics during photodynamic therapy with verteporfin,” Photochem. Photobiol. 74(5), 700 (2001). PHCBAP 0031-8655 10.1562/0031-8655(2001)074<0700:AOTHOP>2.0.CO;2 11723798

[68] Mousavi M. et al., “Photodynamic therapy dosimetry using multiexcitation multiemission wavelength: toward real-time prediction of treatment outcome,” J. Biomed. Opt. 25(06), 063812 (2020). JBOPFO 1083-3668 10.1117/1.JBO.25.6.063812 32246614 PMC7118359

[69] Sunar U. et al., “Monitoring photobleaching and hemodynamic responses to HPPH-mediated photodynamic therapy of head and neck cancer: a case report,” Opt. Express 18(14), 14969 (2010). OPEXFF 1094-4087 10.1364/OE.18.014969 20639983 PMC2964147

[70] Penjweini R. Kim M. M. Zhu T. C., “In-Vivo outcome study of HPPH mediated PDT using Singlet Oxygen Explicit Dosimetry (SOED),” Proc. SPIE 9308, 93080N (2015). PSISDG 0277-786X 10.1117/12.2076441 PMC4437638 25999656

[71] Cottrell W. J. et al., “Irradiance-dependent photobleaching and pain in δ-aminolevulinic acid-photodynamic therapy of superficial basal cell carcinomas,” Clin. Cancer Res. 14(14), 4475–4483 (2008). 10.1158/1078-0432.CCR-07-5199 18628462 PMC2810858

[72] Siddiqui S. A. et al., “Clinical evaluation of a mobile, low-cost system for fluorescence guided photodynamic therapy of early oral cancer in India,” Photodiagn. Photodyn. Ther. 38, 102843 (2022). 10.1016/j.pdpdt.2022.102843 PMC9177774 35367616

[73] Farrell T. J. et al., “Comparison of the in vivo photodynamic threshold dose for photofrin, mono- and tetrasulfonated aluminum phthalocyanine using a rat liver model,” Photochem. Photobiol. 68(3), 394 (1998). PHCBAP 0031-8655 10.1111/j.1751-1097.1998.tb09698.x 9747595

[74] Lilge L. Portnoy M. Wilson B. C., “Apoptosis induced in vivo by photodynamic therapy in normal brain and intracranial tumour tissue,” Br. J. Cancer 83(8), 1110–1117 (2000). BJCAAI 0007-0920 10.1054/bjoc.2000.1426 10993661 PMC2363569

[75] Henderson B. W. Busch T. M. Snyder J. W., “Fluence rate as a modulator of PDT mechanisms,” Lasers Surg. Med. 38(5), 489–493 (2006). LSMEDI 0196-8092 10.1002/lsm.20327 16615136

[76] Sandell J. et al., “A treatment planning system for pleural PDT,” Proc. SPIE 7551, 75510C (2010). PSISDG 0277-786X 10.1117/12.843044 PMC4438758 26005242

[77] Weersink R. A. et al., “Techniques for delivery and monitoring of TOOKAD (WST09)-mediated photodynamic therapy of the prostate: clinical experience and practicalities,” J. Photochem. Photobiol. B 79(3), 211–222 (2005). JPPBEG 1011-1344 10.1016/j.jphotobiol.2005.01.008 15896648

[78] Wang S. et al., “Scalable and accessible personalized photodynamic therapy optimization with FullMonte and PDT-SPACE,” J. Biomed. Opt. 27(8), 083006 (2022). JBOPFO 1083-3668 10.1117/1.JBO.27.8.083006 35380030 PMC8978262

[79] Zhou X. et al., “Photosensitizer dosimetry controlled PDT treatment planning reduces inter-individual variability in response to PDT,” Proc. SPIE 6139, 61390P (2006). PSISDG 0277-786X 10.1117/12.647439

[80] Zhu T. C. Parsai E. I. Orton C. G., “PDT is better than alternative therapies such as brachytherapy, electron beams, or low-energy X rays for the treatment of skin cancers,” Med. Phys. 38(3), 1133–1135 (2011). MPHYA6 0094-2405 10.1118/1.3512802 21520824

[81] “RTOG is the successor to the NCI-funded Radiation Therapy Oncology Group,” https://www.rtog.org/About-Us

[82] Marks L. B. et al., “Use of normal tissue complication probability models in the clinic,” Int. J. Radiat. Oncol. 76(3), S10–S19 (2010). 10.1016/j.ijrobp.2009.07.1754 PMC4041542 20171502

[83] Bentzen S. M. et al., “Quantitative Analyses of Normal Tissue Effects in the Clinic (QUANTEC): an introduction to the scientific issues,” Int. J. Radiat. Oncol. 76(3), S3–S9 (2010). 10.1016/j.ijrobp.2009.09.040 PMC3431964 20171515

[84] Zhou Y. et al., “Mitochondria-targeted photodynamic therapy triggers GSDME-mediated pyroptosis and sensitizes anti-PD-1 therapy in colorectal cancer,” J. Immunother. Cancer 12(3), e008054 (2024). 10.1136/jitc-2023-008054 38429070 PMC10910688

[85] Nkune N. W. et al., “Photodynamic therapy-mediated immune responses in three-dimensional tumor models,” Int. J. Mol. Sci. 22(23), 12618 (2021).1422-0067 10.3390/ijms222312618 34884424 PMC8657498

[86] Pires L. et al., “Photochemical immune stimulation eradicates primary pigmented melanoma and induces long-lasting anti-tumor immunity,” in press. 10.1038/s41416-026-03585-w 42744863

[87] Liu T. W. Gammon S. T. Piwnica-Worms D., “Multi-modal multi-spectral intravital microscopic imaging of signaling dynamics in real-time during tumor–immune interactions,” Cells 10(3), 499 (2021). 10.3390/cells10030499 33652682 PMC7996937

[88] Yu J. H. et al., “Noninvasive and highly multiplexed five-color tumor imaging of multicore near-infrared resonant surface-enhanced Raman nanoparticles *in vivo*,” ACS Nano 15(12), 19956–19969 (2021). ANCAC3 1936-0851 10.1021/acsnano.1c07470 34797988 PMC9012519

[89] Ho T. et al., “A membrane fluidization strategy significantly boosts photodynamic therapy and antitumor immunity of porphyrin-lipid nanoparticles,” ACS Nano Med., in press (2025). 10.1021/acsnanomed.5c00053 PMC12904080 41695020

[90] Azad A. K. et al., “High quantum efficiency ruthenium coordination complex photosensitizer for improved radiation-activated photodynamic therapy,” Front. Oncol. 13, 1244709 (2023). FRTOA7 0071-9676 10.3389/fonc.2023.1244709 37700826 PMC10494715

[r91] Ni K. et al., “Nanoscale metal-organic frameworks for mitochondria-targeted radiotherapy-radiodynamic therapy,” Nat. Commun. 9(1), 4321 (2018). NCAOBW 2041-1723 10.1038/s41467-018-06655-7 30333489 PMC6193046

[r92] Azad A. K. Dinakaran D. Moore R. B., “Radiation Activated Photodynamic Therapy (radioPDT) induces lipid peroxidation and vascular mediated tumor regression of prostate cancer,” Sci. Rep. 15(1), 29299 (2025). SRCEC3 2045-2322 10.1038/s41598-025-14652-2 40789883 PMC12339918

[93] Gu X. et al., “X-ray induced photodynamic therapy (PDT) with a mitochondria-targeted liposome delivery system,” J. Nanobiotechnol. 18(1), 87 (2020). 10.1186/s12951-020-00644-z PMC7288491 32522291

[94] Rodrigues J. A. Correia J. H., “Enhanced photodynamic therapy: a review of combined energy sources,” Cells 11(24), 3995 (2022). 10.3390/cells11243995 36552759 PMC9776440

[95] Clement S. et al., “Mechanisms for tuning engineered nanomaterials to enhance radiation therapy of cancer,” Adv. Sci. 7(24), 2003584 (2020). 10.1002/advs.202003584 PMC7740107 33344143

